# A comparative study of nested-PCR and direct agglutination test (DAT) for the detection of *Leishmania infantum* infection in symptomatic and asymptomatic domestic dogs

**DOI:** 10.1186/s13104-021-05654-0

**Published:** 2021-07-13

**Authors:** Yonas Yimam Ayene, Mehdi Mohebali, Homa Hajjaran, Behnaz Akhoundi, Saeedeh Shojaee, Abbas Rahimi-Foroushani, Mohammad Javad Abbaszadeh Afshar, Z. Zarei

**Affiliations:** 1 Department of Medical Parasitology and Mycology, School of Public Health, Tehran University of Medical Sciences, Tehran, Iran; 2 Centers for Research of Endemic Parasites of Iran (CREPI), Tehran University of Medical Sciences, Tehran, Iran; 3 Department of Medical Parasitology and Mycology, School of Medicine, Jiroft University of Medical Sciences, Jiroft, Iran; 4 Department of Epidemiology and Biostatistics, School of Public Health, Tehran University of Medical Sciences, Tehran, Iran

## Abstract

**Objective:**

Canine visceral leishmaniasis (CVL) is the main source of human visceral leishmaniosis (HVL) in Mediterranean region, including Iran and is spread from domestic dogs to *Phlebotomine* sand flies vectors to humans. To control the transmission of HVL, early and accurate detection of infected dogs is paramount importance despite it remains a confronting challenge. Herein, we evaluated the performance of direct agglutination test (DAT) against gold standard nested polymerase chain reaction (nested-PCR) for CVL diagnosis in symptomatic and asymptomatic domestic dogs from endemic areas of Iran.

**Results:**

Venous blood samples were collected from dogs without clinical signs (n  =  30) and with clinical signs (n  =  35) suggestive of *Leishmania infantum* infection. Among 65 samples examined, *Leishmania* DNA was detected by nested-PCR in 89.23% (58/65). Furthermore, 86.15% (56/65) nested-PCR positive samples were also DAT positive. The results of the DAT sensitivity test were 96.43% and 96.67% in symptomatic and asymptomatic dogs, respectively, while the specificity was 100.00% and 60.00% in symptomatic and asymptomatic dogs, respectively. The results of this study also pointed out substantial concordance between DAT test and nested-PCR method in both symptomatic dogs (Κ  =  0.783; P  <  0.001) and asymptomatic dogs (Κ  =  0.618; P  <  0.001). Thus, DAT represents as a simple and economic tool for initial diagnosis of CVL particularly in endemic areas of the disease.

## Introduction

Visceral leishmaniasis is one of the leading lethal parasitic diseases, with a mortality rate of over 90% of human cases (if left untreated), and domestic dogs are known to be the main resrviour of *Leishmania infantum,* causative agent of human and canine visceral leishmaniasis(CVL) in the South America and Mediterranean basin, including Iran[1–4]. In Mediterranean region, where VL is zoonotic form, the presence of CVL seropositive dogs is strongly associated with human visceral leishmaniasis, highlighting the control of CVL is crucial for bringing down human cases of visceral leishmaniasis [3, 5].

*Leishmania*-infected dogs may exhibit a spectrum of clinical manifestation of the disease, or remain asymptomatic seropositive dogs, depending on the balance between humoral and cellular immune responses [6, 7]. In endemic regions, overt clinical signs were observed in less than 50% of dogs, while most dogs remain asymptomatic for prolonged time and the rate of infectivity of both symptomatic and asymptomatic groups of dogs to sand flies vectors does not significantly differ [8, 9]. Thus, detection, quantification and mapping of CVL infected dogs irrespective of their clinical status are a paramount importance for controlling human visceral leishmaniasis [10].

While different diagnostic tests are available, such as microscopic examination, culture, serology, and molecular methods, the diagnosis of CVL still remains unsatisfactory [11–14]. Culture technique is time-consuming, invasive and labor-intensive, microscopic analysis requires trained laboratory professionals and lacks sensitivity [5, 10]. Molecular method has high rates of sensitivity compared to the aforementioned methods; nonetheless, it is expensive and it requires complex laboratory infrastructure, making it difficult to use in the field settings [15, 16]. Direct agglutination test (DAT) is one of the most widely used diagnosis method for routine laboratory diagnosis and large sero-epidemiological studies in endemic countries like Iran [17]. It doesn’t require expertise, it is cost-effective and appropriate for field use [18–20]. Nevertheless, studies have shown that the production of anti-*Leishmania* antibodies vary depending on the clinical manifestations of the disease and high production of antibodies were evident in symptomatic dogs, so the performance of DAT might vary depending on clinical profiles of dogs [21, 22]. Thus, this study was carried out to evaluate the accuracy of DAT for detection of CVL against nested polymerase chain reaction (nested-PCR) from sera samples of symptomatic and asymptomatic domestic dogs in endemic areas of Iran.

## Main text

### Methods

This study was conducted in the district of Meshkin-Shahr, Northwest Iran, where CVL is endemic [23]. Between April and May 2019, venous blood samples were collected from 65 domestic dogs (30 symptomatic and 35 asymptomatic dogs) and from five apparently healthy negative control dogs, and blood samples were centrifuged for 10 min at 800 rpm within 4 h. And separated serum samples were kept at  − 20 °C before usage and serum samples were used for DAT testing and nested-PCR. Experienced veterinarian evaluated the clinical signs of domestic dogs using 14 clinical signs suggestive of *L. infantum*/*L. chagasi* infection as suggested by Siliva et al. 2017[24], presented in Table 1.

**Table 1:** Clinical signs suggestive of *L. infantum/L. chagasi* infections, according to by Siliva et al. 2017(on score from 0 to 4)

Clinical sign	Score				
	0	1	2	3	4
Apathy	Active	Apathetic			
Injuries by ecctoparasite	Absent	Present			
Weight loss	Absent	Light	Moderate	Intense	Cachexia with loss of movement
Lymphadenomegaly	Absent	Localized	Generalized		
Pale mucous membranes	Absent	Present			
Bleeding	Absent	Present			
Cutaneous signs	Bristles	Absent	Present		
Alopecia	Absent	Present			
Skin lesion	Absence	Presence	Ulcer		
Muzzle depigmentation	Absent	Present			
Nails	Normal	Onychogryphosis			
Muzzle/ear lesion	Absent	Present			
Blepharitis	Absent	Present			
Keratoconjuctivities	Absent	Present			
Mucopurulent	Absent	Present			

Prior to the commencement of this study, the details of the study were thoroughly reviewed and approved by ethics committee in research, School of Health and Paramedical Sciences, Tehran University (Code of Ethics: IR.TUMS.SPH.REC.1399.022).

### Direct agglutination test (DAT)

DAT antigen for native Iranian strain of *L. infantum* was obtained from Department of Medical Parasitology and Mycology, School of Public Health, Tehran University of Medical Sciences, Tehran, Iran. The main phases in the preparation of DAT antigen were mass production of promastigotes *L. infantum* (MCAN/IR/07/Moheb-gh) in RPMI1640 plus 10% fetal bovine serum, trypsinization of the parasites, staining with Coomassie brilliant blue, and fixing with 1.2% formaldehyde [17, 18, 25]. The dogs’ serum samples were examined using DAT for the detection of ant-*Leishmania* antibody in V-shaped micro titer plates. Serum dilution ranging from initial 1:20 to end point titer of 1:20,000 were prepared and 50 µl of antigen suspension was added to each wells. In each plate, negative control well (only antigen) and positive control wells (serum samples with confirmed positive) were prepared for comparison. Afterwards we incubated for 12–18 h in humid room temperature at 21–24 °C and then examined agglutination visually. In comparisons with positive and negative controls, compact blue dots were interpreted as negative, while large diffuse blue mats as a positive [17, 19, 26]. The test results were independently examined by two individuals.

### Nested-PCR

DNA extraction from serum specimens was conducted using FavorPrep™ Tissue Genomic DNA Extraction Mini Kit, in compliance with manufacturer’s instructions. All extracted DNA samples were kept at  − 20 °C until used. A two-step nested-PCR assays using the internal transcribed spacer (ITS) region of the SSU-rRNA genes was targeted for DNA amplification.In the first step, external primer pairs, (R5′ AAACAAAGGTTGTGGGGG3′ and F5 AAACTCCTCTCTGGTGCTTGC3′) was used for PCR amplification. In the second step, internal primer, (F5′AATTCAACTTCGCGTTGGCC3′) and R (5′CCTCTCTTTTTTCTCTGTGC3′) was used. The nested-PCR method was carried out as stated in previous study [27]. PCR products were electrophoresed on 1.5% agarose gel and positive samples were compared with both the DNA ladder and known positive control for identification of *Leishmania* species.

### Data analysis

The statistical analysis was performed using SPSS Statistical software version 21 and a P value less than 0.5 was considered as statistically significant. The sensitivity (SE), specificity, positive predictive (PPV) and negative predictive value (NPV) of DAT test were calculated as per the formula presented below: Sensitivity  =  TP/(TP  +  FN) × 100% (TP: true positive, FN: false negative), Specificity  =  TN/(TN  +  FP) × 100% (TN: true negative, FP: false negative), PPV  =  TP/(TP  +  FP) × 100%, and NPV  =  TN/(TN  +  FN) × 100%). Kappa statistic (K) test with 95% Confidence Interval (CI) was used to assess the agreement between DAT test and nested-PCR. K value of 0.40–0.59, 0.60–0.79, and 0.80–0.90 and above 0.90 were interpreted as weak, moderate, strong and almost perfect agreement, respectively [28].

## Results

The results of DAT and nested-PCR tests for visceral leishmaniosis in dogs with and without clinical signs suggestive of CVL are presented in Table 2. Considering the clinical manifestations of the dogs (n  =  65), 30 were considered as symptomatic (46.15%) and 35 (53.85%) as asymptomatic. Using DAT test, 27 (90.5%) and 31 (88.57%) of symptomatic and asymptomatic dogs were tested positive for CVL, respectively, while the result of nested-PCR showed, 30 (85.71%) asymptomatic and 28 (93.33%) symptomatic dogs were found to be infected with CVL. From 65 samples examined by nested-PCR, *Leishmania* DNA was detected in 89.23% (58/65). In addition, 86.15% (56/65) of nested-PCR positive samples were also DAT positive. Also, Nested-PCR was able to detect Leishmanial DNA in two dogs (one symptomatic and one asymptomatic dogs) that were not able to detect by DAT.

**Table 2:** DAT and nested-PCR results for detection of *L. infantum* infection in symptomatic and asymptomatic dogs from visceral leishmanasis endemic area of Iran, 2019

		Symptomatic dogs (n = 30)			Asymptomatic dogs (n = 35)			All dogs (n = 65)		
		Nested-PCR			Nested-PCR					
		Positive	Negative	Total	Positive	Negative	Total	Positive	Negative	Total
DAT	Positive	27	0	27	29	2	31	56	2	58
	Negative	1	2	3	1	3	4	2	5	7
	Total	28	2	30	30	5	35	58	7	65

Considering nested-PCR as gold standard, SE, SP, PPV, NPV and agreement of DAT test with nested-PCR were provided in Table 3. Substantial agreement between DAT and nested-PCR were observed in both symptomatic (K  =  0.783; P  <  0.001) and asymptomatic dogs (K  =  0.618; P  <  0.001). Greater sensitivity were observed in both symptomatic and asymptomatic dogs, while a relatively reduced specificity was observed in symptomatic dogs. PPV showed high accuracy in both symptomatic and asymptomatic dogs, whereas relatively reduced NPV was observed in symptomatic dogs.

**Table 3:** Evaluation of DAT for detection of CVL in symptomatic and asymptomatic dogs as compared to nested-PCR from visceral leishmanasis endemic area of Iran, 2019

			Symptomatic dogs (n = 30)			Asymptomatic dogs (n = 35)
DAT	SE	96.43%	(Kappa coefficient; P) (K = 0.783; P < 0.001)	SE	96.67%	(Kappa coefficient; P) (K = 0.618; P < 0.001)
	SP	100.00%		SP	60.00%	
	PPV	100.00%		PPV	93.55%	
	NPV	66.67%		NPV	75.00%	

## Discussion

Both symptomatic and asymptomatic dogs could harbor *L. infantum*/*L. chagasi* which are infective for *Phlebotomine* sand flies vectors, thereby infected sandflies could spread *L. infantum/L. chagasi* to humans and other animals [9, 29]. Therefore, accurate and rapid diagnostic method for diagnosis of CVL in both symptomatic and asymptomatic dogs are of paramount importance for visceral leishmaniasis control [4, 10]. Though there are plethora of serological tests for diagnosis of CVL, the sensitivity of these diagnostic methods varies depending on the clinical stage of the disease and the immune status of the host [13, 14]. Studies have pointed out that serological tests have shortcomings in sensitivity, especially in asymptomatic dogs, and consequently could underestimate the rate of *Leishmania* infection [13, 14]. Taking in to account the need for accurate, simple and cheap tests for diagnosis of CVL [18, 26], and scarcity of comparative diagnostic performance studies between serlogical tests and nested-PCR method in endemic areas, this study was carried out to bridge this gap.

In this study, nested-PCR was able to detect Leishmanial DNA in two dogs (one symptomatic and one asymptomatic dogs) that were not able to detect by DAT. Similar to our results, other studies ([30, 31]) have also shown that molecular method able to detect CVL when DAT fail to detect. The better performance of nested-PCR over DAT may be due to its increased sensitivity, which allows it to detect low parasite load [31].

Due to its high sensitivity and easy to perform, DAT is widely used for routine laboratory diagnosis and large scale sero-epidemiological studies in Iran [17, 19]. In this study, DAT test showed high sensitivity (96.43% in symptomatic and 96.67% in asymptomatic dogs). Similar to our studies, a number of studies showed high sensitivity (92–100%) of DAT in symptomatic dogs [26, 31]. Previous studies that assessed the performance of DAT in asymptomatic dogs are scare; nonetheless, a study that evaluated DAT test diagnostic accuracy against real-time PCR demonstrated 98.7% sensitivity [31]. In that study, the result for specificity (83%) in asymptomatic group of dogs was superior to the result observed in our study (60%) [31]. In present study, in asymptomatic dogs, DAT shown to be less specific (60.00%) than other studies, that presented 97% [32] and 91% specificity [33]. The discrepancy in specificity between the present study and the previous studies may related to challenges in understanding the time between disease development and production of antibodies at detectable level, highlighting the need for a more robust molecular method to augment early detection of infection [15].

The result of this study showed substantial concordance between DAT test and Nested PCR method in both symptomatic dogs (K  =  0.783; P  <  0.001) and asymptomatic dogs (K  =  0.618; P  <  0.001). The agreement observed in this study was lower than a study that compared concordance of DAT with real-time PCR which showed 83% in dogs with low anti-leishmanial antibody titer and 97% in dogs with high anti-leishmanial antibody titer [31]. Similarly, another study showed higher agreement (96%) than this study [32]. The divergence in the results of agreement between this studies and previous studies may partly stem from lack of perfect reference standard [34] which result in the use of heterogeneous reference standard. Also, the discrepancy in the agreement may explained by difference in batches and antigen types used.

## Conclusion

DAT showed high sensitivity in detection of CVL in both symptomatic and asymptomatic dogs, pinpointing DAT could equally important for diagnosis of CVL in dogs with and without clinical signs suggestive of the disease especially in endemic areas of Iran. Nested-PCR was able to detect Leishmanial DNA in both symptomatic and asymptomatic domestic dogs when DAT fail to detect. Nonetheless, further studies involving highly sensitive and specific reference standard tests are needed to better elucidate diagnostic performance of DAT.

## Limitations

This research is not, however, devoid of limitations. Given that this study is a cross-sectional investigation, there has been limited knowledge of the diagnostic performance of DAT over time based on clinical and clinical-pathological changes, especially in the symptomatic group of dogs. Also, DAT test performance based on parasite loads has not been evaluated.

## Data Availability

Not applicable.

## Abbreviations

DAT: Direct agglutination test
CVL: Canine visceral leishmaniasis
Nested-PCR: Nested polymerase chain reaction
K: Kappa statistic
SE: Sensitivity
SP: Specificity
PPV: Positive predictive value
NPV: Negative predictive value
